# Current markers for infertility in men

**DOI:** 10.5935/1518-0557.20210013

**Published:** 2021

**Authors:** Jéssica Alves Magalhães, Larissa Sousa Ribeiro, João Paulo Arcelino Rego, Claudia Roberta de Andrade

**Affiliations:** 1 Laboratory of Translational Research, Christus University (UNICHRISTUS), Fortaleza, CE, Brazil; 2 Federal University of São Paulo (UNIFESP), São Paulo, SP, Brazil; 3 Federal Institute of Ceará, Boa Viagem Campus (IFCE). Boa Viagem, CE, Brazil

**Keywords:** male infertility, proteomics, semen quality, diagnostic methods

## Abstract

Male infertility accounts for about 30% of the causes of couple infertility and has become a public health concern. Male infertility may be caused by several factors occurring in isolation or association with several complex syndromes. Despite the importance of semen analysis in the initial investigation of infertility, it has been estimated that 15% of infertile men present normal sperm, a proportion that calls for additional tests to further investigate cases of infertility and accurately determine the factors that alter ejaculate quality. In addition to semen analysis parameters, genetics has been drawing attention. The incorporation of genetic diagnostic methods in the routine practice of andrology laboratories is an important step to further improve assisted reproductive technologies. The present study described the current status of the main methods used in male infertility investigation.

## INTRODUCTION

Fertility is defined as the ability of a couple to establish a clinical pregnancy ^(Zegers-Hochschild *et al*., 2017)^. On the other hand, infertility has been recently considered an important public health problem, characterized by the World Health Organization (WHO) as the failure to attain and maintain pregnancy after twelve months of attempts, without the use of contraceptive methods ^(Agarwal *et al*., 2015^; revised by ^Vander Borght & Wyns, 2018)^.

One in seven couples in developed countries and one in four couples in developing nations suffer from reproductive infertility. Depending on the region, fertility rates may reach 30% and affect more than 180 million people, mostly in developing countries ^(Santoro *et al*., 2016; Mascarenhas *et al*., 2012)^. Men are responsible for 20-30% of infertility cases, but contribute to 50% of the general cases, with male infertility affecting about 10% of couples of reproductive age worldwide and, in many cases, the possibility of treatment ^(Agarwal *et al*., 2015)^.

Infertile males have abnormal spermograms, and etiologies may include environmental, dietary, medical, genetic, and physiological factors ^(Auger *et al*., 2001; Kenkel *et al*., 2001)^. Male infertility is the result of several factors that may occur in isolation or association with several complex syndromes. The causes may be related to anatomical malformations, gametogenesis dysfunctions, immunological disorders, ejaculatory disorders or acquired through exposure to certain environmental agents ^(Kuhtz *et al*., 2014)^.

The cyclic process of germ cells for sperm formation occurs throughout the life of men, allowing them to be fit for reproduction not only in youth (revised by ^Sharma *et al*., 2019)^. During the life of an individual, gametes undergo important processes of proliferation, differentiation and morphofunctional maturation ^(Bernabò *et al*., 2010)^; spermatozoa acquire progressive motility and the ability to undergo training during transit through the epididymis, with changes resulting from alterations in the composition of the epididymal luminal fluid microenviroment. Sperm maturation begins in the head of the epididymis and follows through the body to the proximal tail, causing intense morphological and biochemical changes to ensure the formation of spermatozoa that are capable of recognizing and fertilizing secondary oocytes in the female reproductive tract, while allowing spermatozoa to acquire progressive motility, potential for survival and success in fertilization ^(Oh *et al*., 2006)^.

Sperm is the male germ cell. It is equipped with a tail, or flagellum, that allows motility; the intermediate piece, where primarily the mitochondria and the head are located, contains the genetic material and the acrosome. The acrosome is formed by the Golgi complex and is constituted of digestive enzymes that facilitate sperm penetration in the oocyte membrane, a fundamental process for fertilization ^(Fu *et al*., 2018)^.

Physicochemical alterations in sperm involve variations of the extracellular medium, from the epididymis and seminal plasma to the secretions of the female reproductive tract, which induce the activation of membrane receptors that lead to the transduction of the intracellular signal ^(Gadella, 2008)^. Most of the changes that occur in spermatozoa are mediated by proteins in the fluid of the accessory sexual glands ^(Austin, 1951; Chang, 1951)^.

Once completed, the sperm is capable of recognizing, binding and interacting with the oocyte pellucid zone in order to initiate fertilization ^(Bernabò *et al*., 2010; Rahman *et al*., 2013; 2014)^. These signaling pathways direct the cellular response involving intracellular activation through increased calcium concentration ^(Boni *et al*., 2007)^, change and reorganization of the cytoskeleton ^(Abou-haila & Tulsiani, 2009; Barbonetti *et al*., 2008)^. The plasma membrane and the outside of the acrosome membrane become more unstable and gradually acquire the ability to merge with each other ^(Baker *et al*., 2012; Gadella, 2008; Kwon *et al*., 2015; Park *et al*., 2012; 2013; Rahman *et al*., 2015)^.

This paper aims to analyze the main current aspects of male infertility research, as well as to describe the main genetic alterations related to infertility and the effects of male age on semen quality.

## AGE AND FERTILITY

In recent years, the postponement of the birth of the first child until the parents have reached ages in which fertility or female reproductive capacity is lower has increased the incidence of age-related infertility. Different factors contribute to this age-related issue in both men and women ^(Kehlet *et al*., 2018; Steiner & Jukic, 2016)^.

Although female infertility has been the focus of discussions on reproductive aging, a decline in age-related sperm function and consequently male fertility has been shown due to events such as andropause, poorly defined but related to a decline in testicular function, and gradual reductions in testosterone levels each year ^(McLachlan, 2000)^. Sperm parameters, including semen volume, motility and morphology, deteriorate with age, although reductions in sperm concentration have not been observed ^(Kehlet *et al*., 2018; Oh *et al*., 2006)^.

Some studies evaluated age-related changes in sperm analysis (spermogram), but results have been less than consistent. Environmental factors, chronic diseases and different periods of sexual abstinence are some of the factors that have impaired the selection of individuals for analysis and compromised the conclusions of many studies ^(Kehlet *et al*., 2018; Oh *et al*., 2006)^.

The impact of age is still a reason for disagreement between studies on semen parameters. Older age has been associated with decreased semen volume ^(Cavalcante *et al*., 2008)^, sperm motility ^(Colasante *et al*., 2019)^, motility and concentration ^(Dain *et al*., 2011)^, volume and motility ^(Colasante *et al*., 2019)^, or all of the parameters above ^(Colasante *et al*., 2019)^.

It has been reported that male age also interferes with the gestational outcomes of couples facing difficulties getting pregnant ^(Belloc *et al*., 2008)^. The outcomes of more than 17,000 artificial insemination procedures were analyzed, and decreases in pregnancy rates of 12.3% and 9.3% were observed in men under 30 and men over 45, respectively. A directly proportional relationship between the occurrence of miscarriage and paternal age was also described ^(Belloc *et al*., 2008)^. Despite divergences in studies on male infertility, results have demonstrated the effects of aging on the male reproductive system. However, the impacts of age-related ejaculate quality decreases on male fertility have not been well established ^(Cocuzza *et al*., 2008; Desai *et al*., 2009)^.

In the roster of theories that explain human aging, oxidative stress is currently one of the most widely accepted, since a correlation between aging in humans and the oxidative state of the organism has been demonstrated ^(Gharagozloo *et al*., 2016; Toocheck *et al*., 2016; Ammar *et al*., 2019)^.

## OXIDATIVE STRESS AND SPERM VIABILITY

Reactive oxygen species (ROS) are important for the physiology, capacity and function of sperm development ^(Ammar *et al*., 2019; Agarwal *et al*., 2006)^. Superoxide anion, hydroxyl radicals, and peroxide are known byproducts of normal physiological processes, which excessive production results in hydrogen oxidative stress ^(Gharagozloo *et al*., 2016)^.

Oxidative stress is known as a state in which an oxide-generating system is unbalanced with the antioxidant defense system; it has been related to many diseases, including infertility and/or male subfertility ^(Xavier *et al*., 2019; Dhawan *et al*., 2019; Guz *et al*., 2013)^.

The effects of significant oxidative stress on motility and the prevention of this phenomenon by adding catalase ^(Makler *et al*., 1984)^ indicated the involvement of oxygen overload in sperm motility. This study described the harmful effects of ROS on sperm function, such as lipid peroxidation and DNA damage, observed in several species.

ROS are produced physiologically to maintain cellular processes such as sperm maturation, capacitation and sperm-oval interaction ^(De Lamirande & O’Flaherty, 2008; Rivlin *et al*., 2004)^. However, imbalances in ROS production decrease sperm quality via structural modifications in DNA and membrane lipids ^(Koppers *et al*., 2008; Irvine *et al*., 2000; Aitken & Baker, 2006; Bassiri *et al*., 2020; Beygi *et al*., 2020)^.

On the other hand, ROS are also known to cause significant damage to DNA, both in the mitochondria and in the sperm genome ^(Aitken & Curry, 2011; Sawyer *et al*., 2001)^. Spermatic DNA lesions are in turn linked to low fertilization rates, impaired embryo development, loss of pregnancy and birth of defective children ^(Aitken & Baker, 2006; Quinn *et al*., 2018; Tarozzi *et al*., 2007)^.

## FERTILITY AND GENETIC ABNORMALITIES

In addition to factors related to ejaculate quality, many cases of infertility may be associated with chromosomal anomalies and gene mutations ^(Tournaye *et al*., 2017)^. The human species has relatively common chromosomal abnormalities that result from the loss, gain or abnormal rearrangement of one or more of the 46 chromosomes. Considering these occurrences, there are many molecular and genetic mechanisms involved in reproduction that, when altered, may lead to infertility. Some genetic factors have a clear cause-effect relationship with impaired reproductive function and are part of the diagnosis of infertile males. The factors at play in these cases may be further elucidated with the aid of genetic diagnostics ^(Tournaye *et al*., 2017)^.

**Figure 1:**
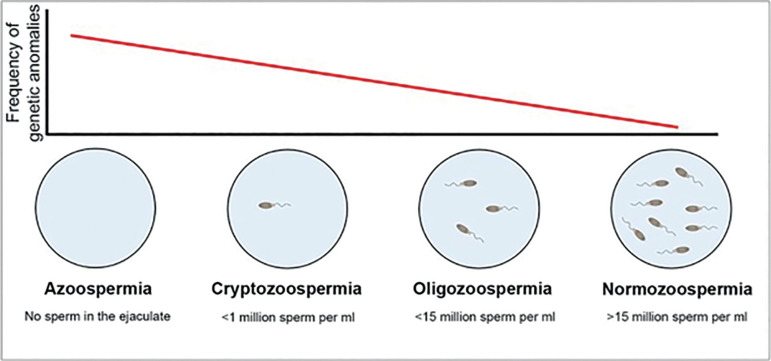
Problems in the number of spermatozoa and correlations with genetic abnormalities. There is a negative correlation between the number of spermatozoa and genetic abnormalities (Adapted from ^Krausz & Riera-Escamilla, 2018)^.

Spermatogenesis disorders related to primary testicular damage might manifest as a variety of semen phenotypes, such as azoospermia (absence of sperm in the ejaculate) and oligospermia (<15 million sperm per ejaculate) (Figure 1). Aspermia is a phenotypic manifestation involving different mechanisms, including interruptions in spermatogenesis in one of the stages of the maturation process and hormone level dysfunctions that affect the androgenization process. This clinical heterogeneity implies the involvement of several genetic or acquired factors ^(Krausz & Riera-Escamilla, 2018)^.

In these cases, specific tests are performed to analyze an individual’s karyotype through cytogenetics tests performed to study the chromosome structure, its pathologies, functions and properties. Due to technological advances, it is possible to use the banding technique in which the chromosome is individually analyzed to identify and recognize structural abnormalities associated with specific genetic syndromes ^(Tournaye *et al*., 2017)^.

The main genetic alterations that lead to male infertility are chromosomal anomalies, including Klinefelter syndrome and XXY mosaicism; 47, XXY. Genetic mutations such as the ones tied to cystic fibrosis and Y chromosome microdeletions may also occur ^(Lanfranco *et al*., 2004)^.

## KLINEFELTER SYNDROME

Klinefelter syndrome is characterized by hypergonadotropic hypogonadism, low testosterone levels, high FSH and LH levels. Subjects with Klinefelter syndrome are traditionally described as infertile due to lack of ability to complete the spermatogenic process that leads to spermatozoid formation ^(Aksglaede *et al.*, 2006)^.

It is characterized by polysomy X, with disomy of chromosome X (47, XXY) as the most frequently observed variant. In 90% of cases, karyotype 47, XXY appears spontaneously when there is non-disjunction of a pair of X chromosomes during meiosis I or II of parental spermatogenesis. The remaining 10% present a mosaic form of the syndrome (46XY/47XXY). The X chromosome comprises about 1100 genes essential to the normal functioning of the testis and brain. Thus, individuals manifest essentially dysfunctions in these two systems ^(Giudice *et al.*, 2019)^. Early diagnosis of this syndrome at a younger age is clearly relevant as it might allow preventive cryopreservation of ejaculated or testicular sperm to preserve fertility and the initiation of preventive therapies for associated non-reproductive health problems ^(Calogero *et al*., 2017)^.

Sperm analysis of patients with Klinefelter syndrome or mosaic Klinefelter syndrome, evaluated by *in situ* hybridization, showed rates of aneuploidy ranging from 2% to 25% and 1.5% to 7.0%, respectively, increasing the probability of passing chromosomal aneuploidies on to the next generations ^(Kovanci *et al*., 2001)^.

## Y CHROMOSOME DELETIONS

The search for Y chromosome microdeletions is performed through molecular tests using the polymerase chain reaction technique. These tests aim to identify the presence of the three AZF regions on the Y chromosome, through probes designed to bind to human DNA and identify the regions of interest ^(Sen *et al*., 2004)^.

The method of *in situ* hybridization of DNA with fluorescence analysis allows the visualization of numerical alterations directly in the chromosome through a fluorescence microscope that analyzes the sequences of DNA marked with fluorochrome, allowing the analysis of a great number of sperm cells, even if the ejaculate contains few gametes. However, this method permits the analysis of numerical changes only ^(Rego *et al*., 2016)^.

Deletions involving the Y chromosome may impair fertility, with 10% azoospermia ^(Stahl *et al*., 2010)^. Considered the smallest chromosome in the human genome (60 million base pairs), the Y chromosome consists mainly of non-recombinant regions, called male-specific region of the human Y chromosome (MSY) ^(Lanfranco *et al*., 2004)^. The Y chromosome is essential for male sexual determination, formation and maintenance of germ cells, with its short arm responsible for testicular development and the long arm responsible for factors involved in spermatogenesis ^(Sen *et al*., 2004)^.

Treatments to reduce sperm DNA damage might also reduce the risk of miscarriage and the risk of implantation failure in intracytoplasmic sperm injection cycles (ICSI), also showing less delay in embryo kinetics ^(Schmid *et al.*, 2007)^.

## SPERM QUALITY AND DNA INTEGRITY

Correlations between age and sperm DNA damage have been described. A study analyzed 1,125 semen samples for DNA fragmentation. The patients were divided into age groups featuring individuals aged 30 and 45 years, < 30 years and >45 years. The group of men aged 45 years presented higher levels of DNA fragmentation than the other groups ^(Moskovtsev *et al*., 2006)^. As in another study evaluating semen parameters DNA fragmentation, chromatin integrity, genetic mutations and chromosomal abnormalities in 97 men aged between 20 and 80 years, the authors observed a positive correlation between age and level of DNA fragmentation ^(Wyrobek *et al.*, 2006)^.

On the other hand, the analysis of semen samples from 140 infertile patients (24-76 years) and 50 men with proven fertility (25-65 years) found no correlation between age and level of DNA fragmentation in any of the groups. To understand these factors, it is important to know the structure of spermatozoa, as well as its process of maturation and training for fertilization ^(Brahem *et al*., 2011)^.

The improvement and wide use of assisted reproductive technologies, especially ICSI, reveals the even greater and more cautious need for the evaluation of sperm nuclear DNA. Spermatozoids with decreased integrity of nuclear DNA, when used to inseminate an egg, may result in fertilization failure or poor prognosis of pregnancy, including early miscarriage. In cases of recurrent miscarriage without an apparent cause, tests to assess the integrity of spermatic DNA chromatin are ordered. This demonstrates that the quality of sperm chromatin plays a fundamental role in the sperm-egg interaction, embryo implantation and division of blastomeres ^(Piasecka *et al.*, 2006)^.

## SPERM QUALITY DIAGNOSTIC METHODS

### SPERMOGRAM

Male fertility analysis was initially required to include an objective evaluation of sperm motility, since there are well-organized protocols showing the lowest standard limits and sperm parameters for ejaculate from normal fertile men ^(Barták, 1977)^. The spermogram is one of the main tests used in semen analysis, along with patient interviews to capture life history ^(Peivandi *et al*., 2019)^.

The following parameters are analyzed in a spermogram: volume, viscosity, pH, color and presence of round cells, sperm, mobility, morphology, concentration and vitality. Other complementary, more specific tests include assessment of fragmentation of sperm DNA, in addition to immunological and molecular tests ^(Clavert *et al*., 1999)^.

These tests comprise the basis of couple infertility investigation ^(Clavert *et al*., 1999)^. Diagnosis usually relies on analyses of sperm concentration, motility and morphology, as well as analysis of the whole ejaculate ^(Peivandi *et al*., 2019)^.

Early fact-finding includes running a spermogram and interviewing the patient to unearth history of infections - particularly sexually transmitted diseases, testicular trauma, varicocele, phimosis, impotence, premature ejaculation, malformations, exposure to radiation and chemical agents, preexisting systemic diseases, habits such as alcoholism and smoking, and prescribed medications ^(Peivandi *et al*., 2019)^.

### SPERM DNA TESTING

Despite the importance of semen analysis in the initial investigation of infertility, it has been estimated that 15% of infertile men present normal spermograms, suggesting that complementary and more specific tests are required for a thorough infertility investigation ^(Aitken *et al*., 2010)^.

Tests that evaluate sperm DNA integrity are of great clinical relevance in the investigation of male infertility. DNA fragmentation is routinely analyzed in andrology laboratories, with the following tests ranking as the most cited in literature and widely available in clinics: sperm chromatin structure assay (SCSA); sperm chromatin dispersion (SCD) test; terminal deoxynucleotidyl transferase dUTP nick end labeling (TUNEL) ^(Fernández *et al*., 2003; Gorczyca *et al*., 1993; Larson *et al*., 2000)^.

SCSA allows the determination of the proportion of sperm susceptible to DNA fragmentation ^(Evenson & Jost, 2000)^. Since it is carried out in the flow cytometer, this test allows the analysis of a great number of cells ^(Zarén *et al*., 2019)^. The SCD test is based on the principle that spermatozoa with fragmented DNA do not produce the characteristic halo when mixed with agarose. It is a technique described as simple and fast, because it does not rely on the intensity of fluorescence and does not require complex and expensive equipment to be run ^(Grèze *et al*., 2019)^.

The TUNEL assay consists of adding, by means of the terminal deoxynucleotidyl transferase enzyme (TdT), modified nucleotides marked with fluorescent molecules to the fragmented strips. The assay identifies fragmentation in both single and double strands. The technique has high sensitivity and specificity, but is deemed complex and less affordable ^(La Vignera *et al*., 2012; Krayevsky *et al*., 2000)^.

### PROTEOMIC ANALYSIS

Sperm cells are among the most highly differentiated cells and are made up of a head with a highly compacted chromatin structure and a large flagellum with a central piece that contains the machinery necessary for movement and the paternal genetic and epigenetic material provided to the oocyte. The whole process of sperm differentiation results in a very peculiar protein composition in the mature sperm cell ^(Thacker *et al*., 2011)^.

The evaluation of the protein profile between sperm of different species has revealed different combinations. This variation in protein arrangement confirms that proteins selected from a certain stage of maturation may not be sufficient for diagnosis or prognosis. However, proteomic approaches have been standardized and may be used for subsequent diagnosis of male fertility ^(Kwon *et al*., 2017)^.

Technological advances in proteomics have made it possible to quantify protein abundance and to distinguish the different functions and state of spermatozoa. In the case of maturation of spermatozoa that come out of the testicles, they need to enter in the head of the epididymis for the maturation process to occur and are stored in the tail, thus producing the motility needed for fertilization. Sperm that have not undergone maturation are considered immature and unable to fertilize an oocyte on their own ^(Belleannee *et al*., 2011)^. In such cases, fertilization can only occur with the help of assisted reproductive technologies such as ICSI ^(Kang *et al.*, 2018)^ and ejaculated sperm versus aspirated sperm in the epididymis ^(Dacheux & Dacheux, 2013)^.

However, few studies have looked into proteomic modifications in spermatozoa during the functional maturation processes. Sperm chromatin compacting reduces its volume, giving sperm an ergonomic shape that facilitates its journey to the egg, protecting it from genotoxic, physical, and chemical factors while ensuring the integrity of the genome. There is evidence that semen proteins modulate sperm function. Correlations between some proteins and the fertility rate of certain species indicate that they are potential molecular markers of reproductive capacity ^(Agarwal *et al*., 2014; du Plessis *et al*., 2011; Boe-Hansen *et al*., 2015; Park *et al*., 2013; Rolland *et al*., 2013)^.

### OXIDATIVE STRESS ANALYSIS

Fertility specialists are exploring the diagnosis of stress in sperm to evaluate the possible use of antioxidants to improve sperm quality ^(Gharagozloo *et al*., 2016)^. Semen plasma contains antioxidants needed for fertilization ^(Atig *et al.*, 2012)^. Oxidative stress is a condition associated with an increase in the rate of cell damage induced by reactive oxygen species ^(Wyck *et al*., 2018)^.

Excessive generation of ROS was found to induce oxidative damage to the plasma sperm membrane, which then loses its ability to respond to essential calcium signaling in the fertilization process ^(Aitken *et al*., 2010)^. It may be experimentally suggested that if normal spermatozoa were artificially exposed to ROS in vitro, they would lose the ability to fertilize, mimicking the *in vivo* situation ^(Aitken & Baker, 2006)^.

Antioxidants have been widely used in subfertile males. Several studies have demonstrated that they contribute positively to sperm count, motility and morphology, and help reduce sperm DNA fragmentation ^(Gharagozloo *et al*., 2016)^.

In treatments using ICSI, it is still unclear whether antioxidants help improve pregnancy and birth rates. High quality studies, including different groups of patients, are needed to elucidate the need for antioxidants in ICSI procedures. Oxidative stress has been established as one of the main causes of male infertility and has been often associated with many diseases that cause infertility. In recent years, protein analysis has been used to characterize the protein profiles of the ejaculate of men with different clinical conditions, such as high oxidative stress ^(Agarwal *et al*., 2014)^.

## CONCLUSION

Further studies are needed to define which factors alter the quality of the ejaculate and the specific parameters affected. Delaying fatherhood is an emerging trend, and this new profile has served as a warning for the possible causes of the negative effects of aging on fertility.

With the evolution of assisted reproductive technologies, it is possible to observe even wider multifactorial causes of infertility and different, more specific shades to male factor infertility. In addition to semen parameters, genetics and the anomalies secondary to germ cell mutations have commanded attention. Individuals with somatic chromosomal anomalies, with an atypical number of chromosomes or structural abnormalities, have a higher probability of infertility, repeat miscarriages, and of or having offspring with severe disabilities.

The incorporation of genetic diagnostic methods in the routine practice of andrology laboratories is an important step to further improve assisted reproductive technologies, minimize the adverse effects of gamete manipulation, and optimize results. During the fertility evaluation process of a couple, careful analysis is warranted to identify potential genetic anomalies and ensure accurate genetic counseling.

Different studies have elaborated on the risks associated with decreases in semen quality and fertility introduced by aging. However, due to the diversity of reported results, additional studies examining the relationship between age and semen quality/fertility are needed before definitive conclusions can be drawn. These studies should include large populations and apply methodological rigor to improve the reliability of results.

In recent years, proteomic analyses have been used to characterize the protein profiles of the ejaculate of men with different clinical conditions. Recent advances in proteomic techniques, especially in two-dimensional polyacrylamide gel electrophoresis and mass spectrometry, have enhanced the study of spermatozoa and sperm proteins. One of the advantages of gel electrophoresis is that the technique allows the identification of various specific sperm proteins. Proteomics has also provided additional insight into the role of the proteins involved in sperm processes and the differentiation between normal and abnormal states. In addition, studies on sperm proteome demonstrated the importance of post-translational modifications and their ability to cause physiological changes in sperm function. The recent advances in diagnostic techniques may provide information on sperm function and dysfunction and be implemented in human reproduction clinics to identify and characterize the damages that cause male infertility.
